# LARP3 inhibits the apoptosis of hepatocellular carcinoma via the ROS/PI3K/c-Fos axis

**DOI:** 10.1371/journal.pone.0317454

**Published:** 2025-01-17

**Authors:** Lin Zhu, Qianqian Meng, Weiyi Qian, Weiting Shao, Yuyue Lu, Shuai Jin, Afei Zhang, Shuang G. Yan, Jingtao Lu

**Affiliations:** 1 School of Life Sciences, Anhui Medical University, Hefei, Anhui, China; 2 School of Pediatrics, Xinjiang Medical University, XinJiang, China; 3 Department of Orthopaedic Surgery, The First Affiliated Hospital of Anhui Medical University, Hefei, Anhui, China; Xiangya Hospital Central South University, CHINA

## Abstract

Primary hepatocellular carcinoma (PHC) is the sixth most common cancer and the third leading cause of cancer death worldwide. Hepatocellular carcinoma (HCC) accounts for 75%-85% of PHC. LARP3 is aberrantly expressed in multiple cancers. We found that it is significantly highly expressed in the liver cancer tissues of HCC patients, but the exact role and specific mechanism of this abnormal expression are not yet clear. In this study, through bioinformatics analysis, we concluded that LARP3 expression is associated with a poor prognosis for patients with HCC. Through cellular experiments such as gene editing and phenotypic functions, we found that LARP3 promotes the occurrence and development of HCC and inhibits apoptosis. Finally, through biological means such as RNA sequencing, flow cytometry, western blotting, and the construction of a subcutaneous tumorigenesis model in nude mice, we concluded that inhibition of HCC apoptosis by LARP3 is related to LARP3 negatively regulating ROS level and inhibiting the PI3K/c-Fos/apoptosis axis. This study will provide potential targets for the treatment of HCC.

## Introduction

Primary hepatic cancer (PHC) is the sixth most common cancer and the third leading cause of cancer death globally, with a 5-year relative survival rate of less than 20% [1]. PHC includes hepatocellular carcinoma (HCC), which accounts for the vast majority [2], and intrahepatic cholangiocarcinoma [3], as well as other rare types of liver cancer. Cirrhosis [4] and hepatitis [5] viruses are important prerequisites for the lesion of HCC. Long-term heavy drinking [6] and non-alcoholic fatty liver disease [7] are also important risk factors for HCC, increasing the risk of developing hepatocellular carcinoma. At the same time, some chemicals in the environment, such as aflatoxin, can also cause damage to the liver [8]. The radical treatment method for HCC is surgery, including hepatectomy and liver transplantation surgery [9]. Intra-arterial therapy, ablation, and radiotherapy are also important means for treating HCC [10]. Due to the fact that the pathogenesis of HCC is not very clear and the inducing causes are complex and cumbersome, most HCC patients are already in the advanced stage when they seek medical treatment [11]. Therefore, more comprehensive and effective treatment approaches and predictive biomarkers with therapeutic stratification benefits are still needed.

The Small RNA Binding Exonuclease Protection Factor La (LARP3), which is a member of the La-related protein family, shows important cellular regulatory functions and has been reported to be associated with various diseases, including cancer [12]. In addition to the basic LaM (winged helix fold)-RRM (RNA recognition motif) 1 composition, LARP3 also includes RRM2 [13]. NMR studies have shown that RRM2 may be involved in the binding of La to the hepatitis C IRES (internal ribosome entry site), suggesting that LARP3 is involved in carcinogenesis and cancer progression [14]. The expression and activity levels of LARP3 vary among different cancer types [15], indicating that it may potentially serve as a biomarker for the diagnosis, prognosis assessment, and screening of treatment targets in cancer.

The abnormal generation of reactive oxygen species (ROS) has a very close relationship with the occurrence and development of cancer and is an important factor in tumorigenesis, development and recurrence [16]. When ROS is overly produced or the cellular clearance ability is reduced, ROS will accumulate in the cells, triggering a series of oxidative stress responses [17]. ROS has an important second messenger function and participates in the regulation of many fine pathways in the cell, including inducing autophagy and apoptosis [18, 19]. It is reported that the accumulation of ROS in tumor cells can cause apoptosis, thereby inhibiting the proliferation and spread of tumors [20]. In addition, ROS can induce relevant inflammatory responses at the tumor site and increase the immunogenicity of the tumor [21].

## Materials and methods

### Data acquisition

Transcriptome sequencing and clinical pathological data were retrieved from the TCGA dataset (https://portal.gdc.cancer.gov/), and LIHC (Liver Hepatocellular Carcinoma) data were downloaded. The differential expression of LARP3 protein in normal liver and HCC tissue was analyzedusing immunohistochemistry (IHC) data from the Human Protein Atlas database (HPA, https://www.proteinatlas.org/). In addition, we performed LARP3 pancancer expression analysis using TIMER 2.0 (http://timer.cistrome.org/). Transcriptome sequencing and clinical pathological data were retrieved from the TCGA dataset (https://portal.gdc.cancer.gov/), and LIHC (Liver Hepatocellular Carcinoma) data were downloaded. The differential expression of LARP3 protein in normal liver and HCC tissue was analyzedusing immunohistochemistry (IHC) data from the Human Protein Atlas database (HPA, https://www.proteinatlas.org/). In addition, we performed LARP3 pancancer expression analysis using TIMER 2.0 (http://timer.cistrome.org/).

### Independent prognostic and clinical characteristic analysis of gene LARP3

The patients were divided into high-and low-expression groups according to the median expression level of gene LARP3. Survival analysis was performed using Kaplan-Meier and log-rank tests. We also assessed the relationship between LARP3 and clinical characteristics such as age, sex, stage, grade, and TNM stage. In addition, univariate and multivariate Cox analyses were performed for LARP3 and clinical characteristics to determine independent prognostic indicators. Analyses were performed using R software’s “survival” and “survminer” packages (v 4.0.2). *p* <0.05 was considered significant.

### Cell culture and reagents

The L02, Li-7 and SUN449 cells were cultured in RPMI-1640 medium supplemented with 10% FBS and 1% penicillin/ streptomycin at 37°C in a humidified atmosphere with 5% CO_2_. The HepG-2, Hep3B and HEK-293T cells were cultured in DMEM medium supplemented with 10% FBS and 1% penicillin/streptomycin at 37°C in a humidified atmosphere with 5% CO_2_.

NAC (N-Acetyl-L-cysteine, a ROS scavenger) and 740Y-P (an effective and cell-permeable PI3K activator) were purchased from TargetMol (Shanghai, China).

### Plasmid and primer information

Overexpression was achieved using the pLVX-Puro plasmid, knockdown was achieved using the pLKO.1-Puro lentiviral interference plasmid, and transient overexpression was achieved using the pCDNA3.1 plasmid. The sequence information of the LARP3 and c-Fos primers is in S1 Table.

### Quantitative real-time PCR (qRT-PCR)

Total RNA was extracted using a Trizol Reagent (Genesand, China). The purity and concentration of RNA were determined using a NanoDrop 2000 spectrophotometer (Thermo Fisher Scientific, Waltham, ME, USA). Reverse transcription was performed using Hifair RIII1st Strand cDNA Synthesis SuperMix (Yeasen, Shanghai, China) for cDNA synthesis. Realtime quantitative PCR was per-formed using Real-time PCR instrument LightCycler 480II (Roche, Shanghai, China). GAPDH was employed as the reference gene. Relative gene expression was calculated using 2^-ddCt^ method. Sequences of PCR primers were listed in S1 Table (Qingke Biotech, China).

### Western blotting

Total protein was extracted from cells in each group using RIPA lysate (Beyotime Biotechnology, China). The protein concentration was measured by the Bradford (Biosharp, Beijing, China) method. Then, the proteins were separated by SDS-PAGE. After the target proteins were transferred to the nitrocellulose filter (PVDF, Merck, China) membrane, it was blocked with 5% skimmed milk at room temperature for 90 min. Next, these membranes were incubated with primary antibodies such as LARP3 (1: 2000, HUABIO, Hangzhou, China), GAPDH (1: 10000, Proteintech, Wuhan, China), Caspase3 (1: 1000, ZEN BIO, China), Cleaved-Caspase3 (1: 2000, ZEN BIO, China), BCL2 (1: 1000, Coherion, China), BAX (1: 1000, Coherion, China), PI3K (1:2000, ImmunoWay, USA), p-PI3K (1:2000, ImmunoWay, USA), c-Fos (1: 500, HUABIO, Hangzhou, China), and p-c-Fos (1:2000, ImmunoWay, USA) overnight at 4°C. Subsequently, these membranes were incubated with the corresponding secondary antibody for 90 min at room temperature. The membranes were exposed using an imaging system (Tanon, Shanghai, China).

### Cell proliferation, colony formation, migration, and invasion assay

The indicated HCC cells were seeded in 96-well plates at a density of 2 × 10^3^ cells/well, and proliferation was detected using the CCK-8 kit (TargetMol Shanghai, China) according to the manufacturer’s instructions. For the colony formation assay, 1 × 10^3^ cells were seeded in a 6-well plate and kept with 10% FBS DMEM for 2 weeks. The colonies were fixed with methanol for 30 min and subsequently stained with 0.1% crystal violet for 20 min. Clones with more than 50 cells were defined as positive. For cell migration, in the 12-well plate, cells were seeded at a density of 2.5 × 10^5^ per well. After cell adhesion has been completed, use a 200 µL yellow pipet tip to scratch along the horizontal line behind the plate against the vertical ruler. The tip of the pipet should be kept vertical during the scratching process to avoid tilting. Then, wash twice with PBS buffer to remove floating cells in the scratch. Subsequently, replace the medium with 2% FBS and place the culture plate in the cell culture incubator for culture for 48 h. During this period, observe the width of the scratch with a microscope and take pictures to record. For Transwell invasion, we diluted the matrix gel on ice according to the ratio of Matrigel gel to serum-free medium as 1:6, added 60 µL to each chamber after evenly mixing, to avoid bubble generation, and then placed the chamber in the cell culture incubator for incubation for 3 h for later use. Then we put the chamber in the 24-well plate (filled with 650 µL 20% FBS DMEM/well), and seeded 4 × 10^4^ HCC cells (suspended with 200 µL serum-free DMEM) in the upper chamber. After incubation for 36 h, the invaded cells were fixed with methanol and stained with crystal violet. The migrated and invaded cells were captured and calculated using microscope.

### Cell apoptosis analyses by flow cytometry

Seed the cells at 5 × 10^5^ cells / well in the six-well plate. According to the experimental design, treat with NAC (0.25 mM, 24 h; Yeasen, Shanghai, China) and 740 Y-P (20 µM, 24 h; Yeasen, Shanghai, China), and use the Annexin V-FITC/PI apoptosis kit (Yeasen, Shanghai, China) to stain according to the instructions, and then immediately analyze the samples by flow cytometry.

### Reactive oxygen species (ROS) assay

Measurement of intracellular ROS was performed using DCFH-DA (Yeasen,Shanghai, China). Cells were incubated with 10 µM DCFH-DA diluted in DMEM medium for 30 min at 37°C in the dark. Then use flow cytometry to detect the fluorescence intensity.

### In vivo tumourigenesis assays

Nude mouse rearing conditions: Raised in a SPF laboratory environment. The relative humidity is maintained at 40%-60%, and the room temperature is 26–28°C. Under normal lighting hours, the bedding is treated with ultraviolet sterilization. Both food and water are sterilized by high-pressure steam. When euthanasia is performed, sodium pentobarbital is used for anesthesia at a dose of 50 mg/kg to reduce pain. Five-week-old male BALB/c nude mice were randomly divided into two groups (n = 5 / group). The two groups of mice were injected subcutaneously under the armpit with 5 × 10^6^ HepG-2 cells transfected with the empty vector or the LARP3 knockdown vector respectively. The weight of the mice was weighed and recorded every seven days. After 28 days of vaccination, the mice were decapitated to sacrifice, the subcutaneous tumors were separated, weighed and recorded, and the photographic equipment was used to take image materials. The diameter of subcutaneous tumors in nude mice was measured using a vernier caliper. Then the tumor tissue could be fixed with 4% paraformaldehyde for later analysis.

### Statistical analysis

The survival rates patients with HCC were calculated by Kaplan-Meier and log-rank analyses, and prognostic factors were evaluated using the Cox regression model. After the cell clone formation assay and scratch assay were quantitatively processed by Image J, statistical analysis was performed using GraphPad Prism 8.0. For the rest of the statistical charts, statistical analysis was also carried out using GraphPad Prism 8.0. All experiments were performed at least three times. Student’s test, one-way or multi-way analysis of vaiance(ANOVA) were used for statistical comparisons between experimental groups. Data are presented as means ± standard deviation (SD). *p* <0.05 was considered as statistically significant.

## Results

### 1. LARP3 is negatively correlated with the prognosis of HCC

**Fig 1:**
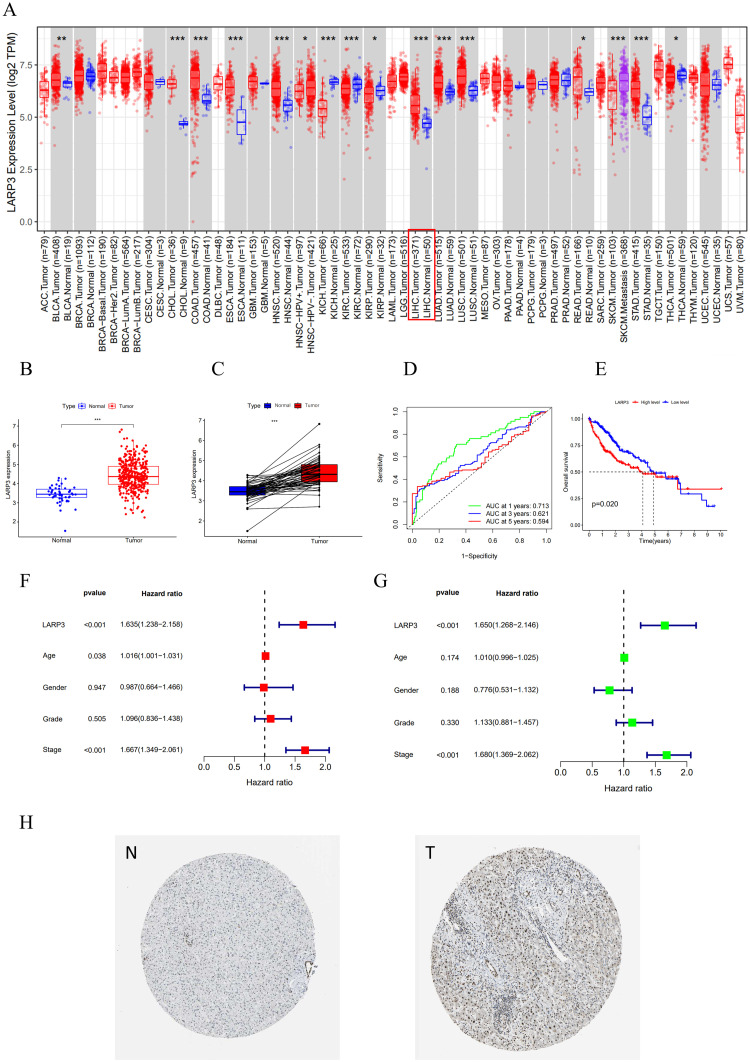
LARP3 is negatively correlated with the prognosis of HCC. A:The expression of LARP3 mRNA in pan-cancer tissues in the TCGA database. B:Box plot of the expression of the LARP3 gene in HCC tissues in the TCGA (N = 50, T = 374). C: Paired box plot of the expression of the LARP3 gene in HCC tissues in the TCGA (N = 50, T = 50) database. D: The K-M survival curve of LARP3 in HCC in the TCGA database. E: The ROC curve of the expression of LARP3 in the HCC database in the TCGA database for 1 year (0.713), 3 years (0.621), and 5 years (0.594). F and G: Multivariate and univariate Cox regression analysis of LARP3 in HCC. H: Immunohistochemical analysis of LARP3 in the HPA database (https://www.proteinatlas.org/), where N represents immunohistochemistry of normal liver tissue samples and T represents immunohistochemistry of samples from patients with hepatocellular carcinoma.

Abnormal expression of LARP3 was observed in multiple different types of tumors, including liver cancer (Fig 1A). The research group chose HCC (LIHC in Fig 1A) for further study. The box plot (Fig 1B) and the paired box plot (Fig 1C) showed that the expression of LARP3 mRNA in cancer tissues was significantly higher than that in normal tissues. Kaplan-Meier survival analysis showed the expected result that the prognosis of the high-expression group was significantly worse than that of the low-expression group (Fig 1D). The receiver operating characteristic curve suggested that LARP3 was related to the prognosis of HCC (Fig 1E). The results of independent prognostic analysis showed that the p-values of LARP3 in both univariate and multivariate Cox regression analyses were significant, suggesting that LARP3 could serve as an independent prognostic factor for HCC independently of other clinical traits (Fig 1F and 1G). Immunohistochemical staining in the HPA database showed that the expression of LARP3 in HCC tissues was significantly higher than that in normal tissues (Fig 1H). To sum up, LARP3 is closely related to HCC and can predict the prognosis of HCC patients to a certain extent.

### 2. Construction of stable LARP3 knockdown and overexpression cell lines

Compared to L02 (a normal hepatocyte line), LARP3 mRNA and protein expression levels were generally highly expressed in HCC cell lines (S1A and S1B Fig). The research group used the lentiviral expression system to establish cell lines to overexpress or knockdown LARP3. The research group knocked down the HCC cell lines HepG-2 and Li-7 with high and moderate expression (S1C–S1F Fig) and overexpressed LARP3 in the moderately expressed cell line Li-7 (S1G and S1H Fig).

### 3. LARP3 promotes the proliferation, migration and invasion of HepG-2 and Li-7 cells

**Fig 2:**
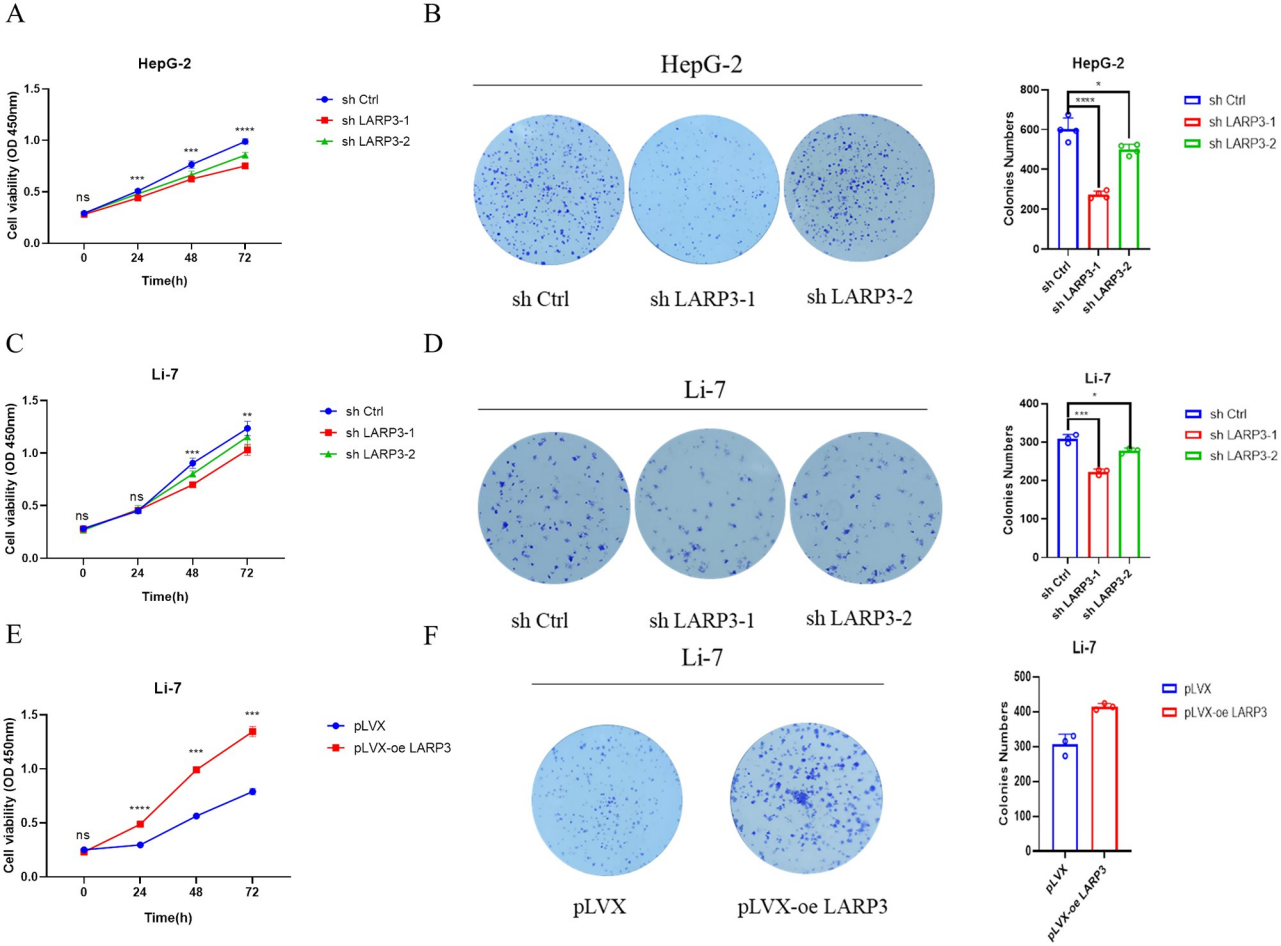
LARP3 promotes the proliferation of HepG-2 and Li-7 cells. A-B: CCK-8 and colony formation assay to detect the proliferation ability of HepG-2 cells with knocked-down LARP3. C-D: CCK-8 and colony formation assay to detect the proliferation ability of Li-7 cells with knocked-down LARP3. E-F: CCK-8 and colony formation assay to detect the proliferation ability of Li-7 cells with overexpressed LARP3. The data are presented in the form of X±s, from 3 independent experiments with similar results. * *p*<0.05, **p <0.01, ***p <0.001, ****p <0.0001.

**Fig 3:**
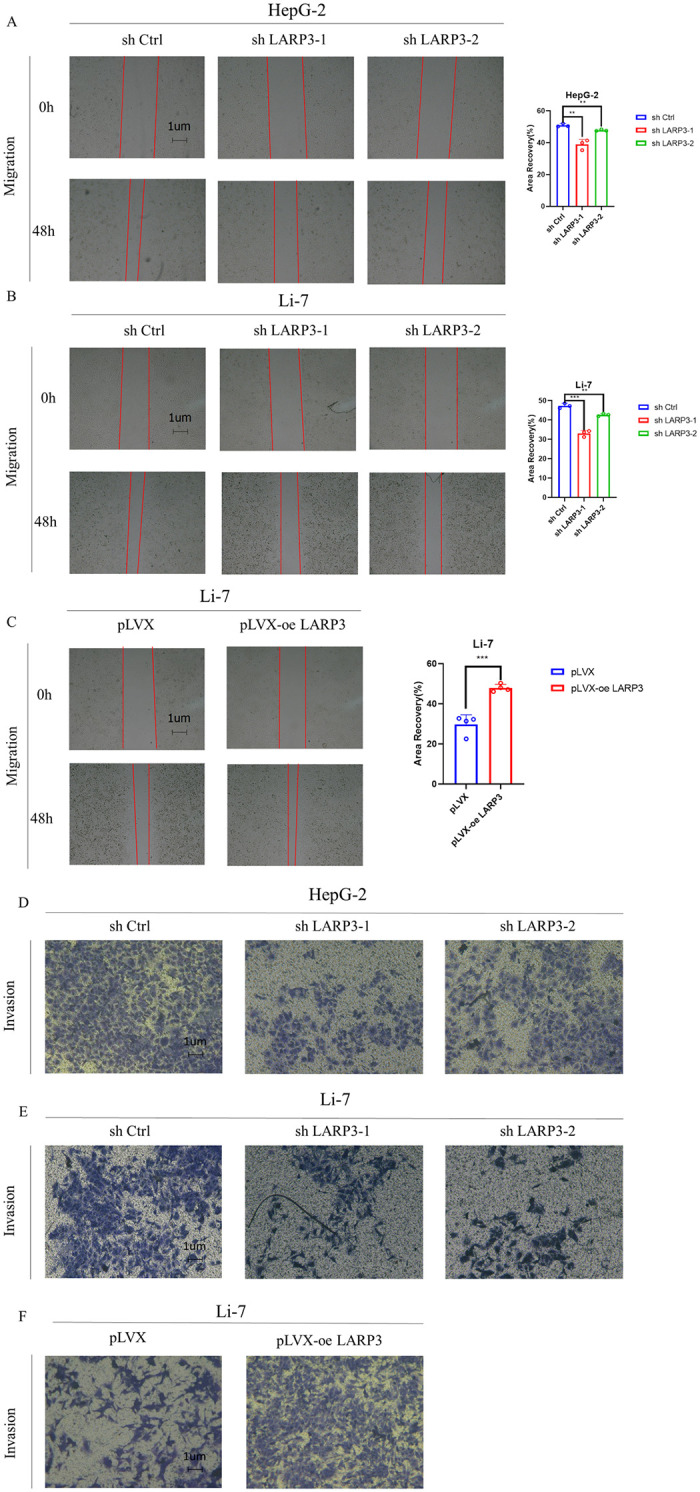
LARP3 promotes the migration and invasion of HepG-2 and Li-7 cells. A-B: Scratch assay to detect the migration ability of HepG-2 and Li-7 cells with knocked-down LARP3. C: Scratch assay to detect the migration ability of Li-7 cells with overexpressed LARP3. D-E: Transwell assay to detect the invasion ability of HepG-2 and Li-7 cells with knocked-down LARP3. F: Transwell assay to detect the invasion ability of Li-7 cells with overexpressed LARP3. The data are presented in the form of X±s, from 3 independent experiments with similar results.*p <0.05, **p <0.01, ***p <0.001.

The proliferation ability of HepG-2 and Li-7 cells was detected by CCK-8 and colony formation assays. The results showed that compared with the control group, knockdown of LARP3 could inhibit the proliferation ability of HepG-2 and Li-7 cells, while the overexpression of LARP3 promoted this ability (Fig 2A–2F). The results of the scratch assay (Fig 3A–3C) showed that compared with the control group, knockdown of LARP3 could inhibit the migration ability of cells, while the overexpression of LARP3 promoted this ability. In the Transwell assay, the invasion ability of HepG-2 and Li-7 cells with knockdown of LARP3 was weakened, and this ability was enhanced with the overexpression of LARP3 (Fig 3D–3F).

### 4. Knockdown of LARP3 promotes apoptosis in HepG-2 cells by down-regulating c-Fos

**Fig 4:**
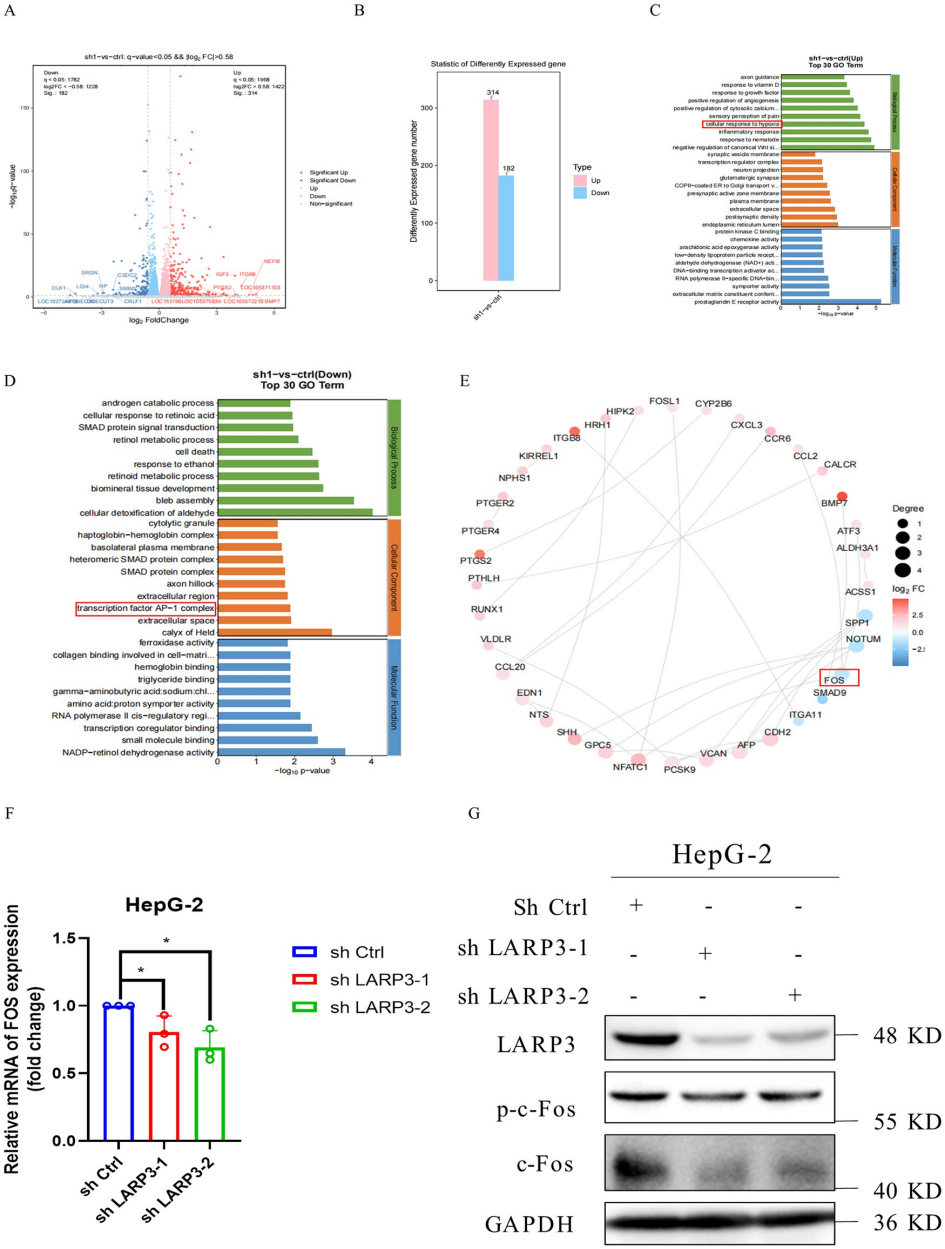
Knockdown of LARP3 promotes apoptosis in HepG-2 cells by down-regulating c-Fos. A: Volcano plot of differentially expressed genes in HepG-2 cells with knocked-down LARP3. B: Statistical chart of differentially expressed genes in HepG-2 cells with knocked-down LARP3, *q value <0.05; |*log*2*FC*| >0.58. C-D: RNA-seq GO functional analysis (Top 30). E: Network map of differentially expressed genes (Top 30). F: RT-qPCR to detect the expression of c-Fos mRNA in HepG-2 cells with knocked-down LARP3. G: Western blot to detect the expression of c-Fos protein in HepG-2 cells with knocked-down LARP3. The data are presented in the form of X±s from 3 independent experiments with similar results. *p <0.05.

The results of transcriptome high-throughput sequencing (RNA seq) showed that after knockdown of LARP3, the expression of 314 genes was significantly upregulated, and 182 genes were significantly downregulated (Fig 4A and 4B). Through GO functional analysis, it can be known that after knockdown of LARP3, the cellular response to hypoxia was upregulated, indicating that cancer cells were more likely to accumulate ROS when facing the hypoxic environment (Fig 4C). At the same time, the research group also found that the expression of the transcription factor AP-1 complex was significantly downregulated (Fig 4D). The AP-1 complex is formed by the interaction between members of the c-Jun and c-Fos protein families in various forms of homo- or hetero-dimers, and plays an important role in the transcriptional regulation of cells [22, 23]. Some studies have shown that the AP-1 complex is associated with the occurrence and development of cancer, and can affect the proliferation, differentiation, migration, invasion and apoptosis of cancer cells by regulating downstream target genes [24]. Among them, c-Fos, as a common proto-oncogene, has aroused the interest of the research group, and it has been proposed in previous studies to have the ability to inhibit apoptosis and promote cell proliferation and migration [25]. Subsequently, the research group found that the mRNA expression level of the c-Fos gene in HepG-2 cells was significantly downregulated after knockdown of LARP3 among the 182 downregulated genes (Fig 4E). In order to verify the sequencing results, the research group verified the expression of c-Fos in HepG-2 cells after knockdown of LARP3 by RT-qPCR and Western Blot (Fig 4F and 4G).

### 5. Knockdown of LARP3 upregulates the ROS level in HepG-2 cells and enhances their sensitivity to hydrogen peroxide

**Fig 5:**
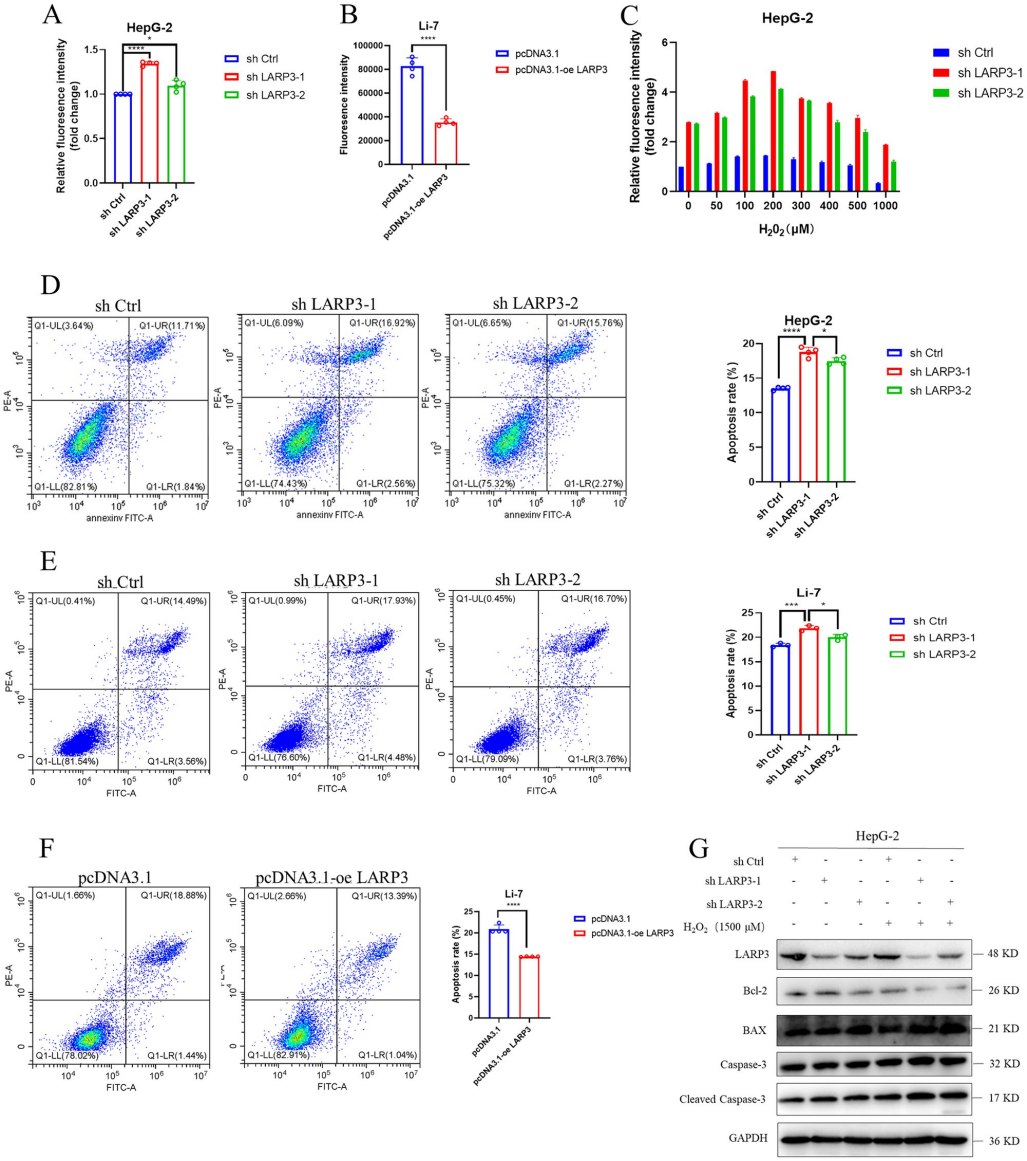
Knockdown of LARP3 upregulates the ROS level in HepG-2 cells and enhances their sensitivity to hydrogen peroxide. A: Microplate reader to detect the ROS level of HepG-2 cells with knocked-down LARP3. B: Flow cytometry to detect the ROS level of Li-7 cells with overexpressed LARP3. C: Changes in the ROS level of HepG-2 cells under the stimulation of different concentrations (0–1000 μM) of H_2_O_2_. D-E: Flow cytometry to detect the apoptotic level of HepG-2 and Li-7 cells with knocked-down LARP3. F: Flow cytometry to detect the apoptotic level of Li-7 cells with overexpressed LARP3. G: Western Blot to detect the changes of apoptosis pathway-related proteins BAX, Bcl-2 and Cleaved Caspase-3 in HepG-2 cells. The data are presented in the form of X±s from 3 independent experiments with similar results. **p <0.05, ***p <0.01, ****p<0.001.

The research group detected the changes in the ROS level of HepG-2 cells after knockdown of LARP3 (Fig 5A), and the results showed that the ROS level of the cells increased after knockdown of LARP3. While after overexpression of LARP3, the ROS level of Li-7 cells decreased (Fig 5B). In order to detect the relationship between LARP3 and the ROS level, the research group constructed an H_2_O_2_ (hydrogen peroxide) oxidative stress model of HepG-2 cells. HepG-2 cells were stimulated with different concentrations of H_2_O_2_ (0–1000 µM). The results showed that after knockdown of LARP3, HepG-2 cells were more sensitive to the stimulation of H_2_O_2_ and more prone to accumulate ROS (Fig 5C). It can be known from the flow cytometry results (Fig 5D–5F) that the apoptosis level of cells increased after knockdown of LARP3; while after overexpression of LARP3, the apoptosis level decreased. The research group treated HepG-2 cells with a high concentration of H_2_O_2_ (1500 µM) and detected the apoptosis pathway-related proteins by Western Blot. The results showed that the apoptosis pathway was significantly activated, and the apoptosis level of HepG-2 cells with knockdown of LARP3 was significantly higher than that of the control group (Fig 5G).

### 6. ROS/PI3K mediates the downregulation of c-Fos by knockdown of LARP3 to promote apoptosis

**Fig 6:**
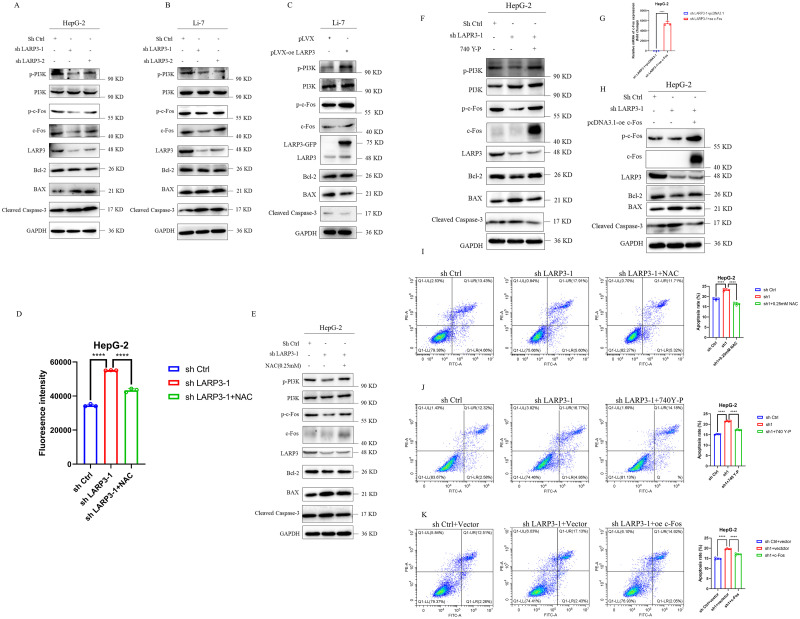
ROS/PI3K mediates the downregulation of c-Fos by knockdown of LARP3 to promote apoptosis. A: Western Blot to detect the PI3K / c-Fos / apoptosis pathway-related proteins in HepG-2 cells with knocked-down LARP3. B: Western Blot to detect the PI3K / c-Fos / apoptosis pathway-related proteins in Li-7 cells with knocked-down LARP3. C: Western Blot to detect the PI3K / c-Fos / apoptosis pathway-related proteins in Li-7 cells with overexpressed LARP3. D: HepG-2 cells with knocked-down LARP3 were treated with 0.25 mM NAC, and the ROS level was detected by flow cytometry. E: HepG-2 cells with knocked-down LARP3 were treated with 0.25 mM NAC, and Western Blot was used to detect the PI3K / c-Fos / apoptosis pathway-related proteins after NAC treatment. F: HepG-2 cells with knocked-down LARP3 were treated with 20µM 740 Y-P, and Western Blot was used to detect the PI3K / c-Fos / apoptosis pathway-related proteins after 740 Y-P treatment. G: RT-qPCR to detect the expression level of c-Fos mRNA. H: Western Blot to detect the apoptosis pathway-related proteins downstream of HepG-2 cells after overexpressing c-Fos. I: Flow cytometry to detect the apoptotic level of HepG-2 cells after NAC treatment. J: Flow cytometry to detect the apoptotic level of HepG-2 cells after 740 Y-P treatment. K: Flow cytometry to detect the apoptotic level of HepG-2 cells after overexpressing c-Fos. The data are presented in the form of X±s from 3 independent experiments with similar results. **p<0.05, ***p <0.01, ****p <0.001.

The research group consulted relevant literature and discovered that the downstream classical pathway PI3K of ROS has a regulatory effect on c-Fos [26]. After knockdown of LARP3, the accumulation of ROS inhibited the phosphorylation of PI3K and the expression of c-Fos, thereby promoting cell apoptosis; while the opposite result was obtained after overexpression of LARP3 (Fig 6A–6C).

In order to clarify the upstream and downstream relationship between the pathways, the research group designed a rescue and restoration experiment for verification. Firstly, on the basis of knockdown of LARP3, the research group used the ROS scavenger NAC for treatment to reduce its ROS level (Fig 6D). The results of Western Blot and flow cytometry showed that after NAC treatment, the apoptosis level of the knockdown strain was also suppressed to a certain extent (Fig 6E and 6I). Subsequently, on the basis of knockdown of LARP3, the research group added the PI3K activator 740Y-P for treatment and found that PI3K was significantly activated. The results of Western Blot and flow cytometry indicated that after the restoration of PI3K phosphorylation, the apoptosis level of the knockdown strain was suppressed (Fig 6F and 6J). Finally, on the basis of knockdown of LARP3, the research group overexpressed c-Fos (Fig 6G), and the detections of Western Blot and flow cytometry showed that the downstream apoptosis pathway was suppressed (Fig 6H and 6K).

### 7. Knocking down LARP3 inhibits the growth of subcutaneous tumors in nude mice

**Fig 7:**
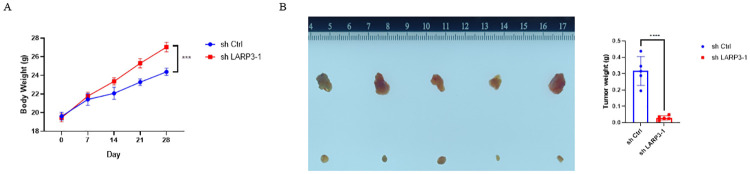
Knocking down LARP3 inhibits the growth of subcutaneous tumors in nude mice. A: The relationship between the body weight of nude mice and time after subcutaneous injection of HepG-2 cells. B: The tumor pictures and the columnar statistical chart of tumor weight of subcutaneous tumorigenesis in nude mice. The data are presented in the form of X±s from 3 independent experiments with similar results. **p <0.05, ***p <0.01, ****p <0.001, *****p <0.0001.

The research group injected HepG-2 cells with knockdown of LARP3 subcutaneously into nude mice, and at the same time set up a control group. The mice were weighed every 7 days, and the mice were sacrificed 28 days after inoculation (Fig 7A). The subcutaneous tumor tissues were dissected and weighed. The maximum diameter of the tumor is 7.3 mm. The results showed that compared with the control group, the proliferation ability of HepG-2 cells with knockdown of LARP3 was significantly weaker than that of the control group HepG-2 cells (Fig 7B).

## Discussion

HCC is the most common type of PHC, and multiple factors are closely related to the occurrence of HCC, including chronic hepatitis B and hepatitis C virus infections, alcoholic liver disease, non-alcoholic fatty liver disease, aflatoxin exposure, genetic factors, etc [27]. HCC has a relatively high incidence and mortality rate globally, and its incidence shows regional differences and may change over time [28]. Its molecular biological mechanism involves a series of gene mutations, abnormal expressions and dysregulation of signal pathways, such as changes in the p53 gene [29], Wnt/β-catenin pathway [30], etc. HCC usually undergoes a process from liver lesions to malignant transformation, and understanding its progression mechanism is crucial for early diagnosis and therapeutic intervention.

**Fig 8:**
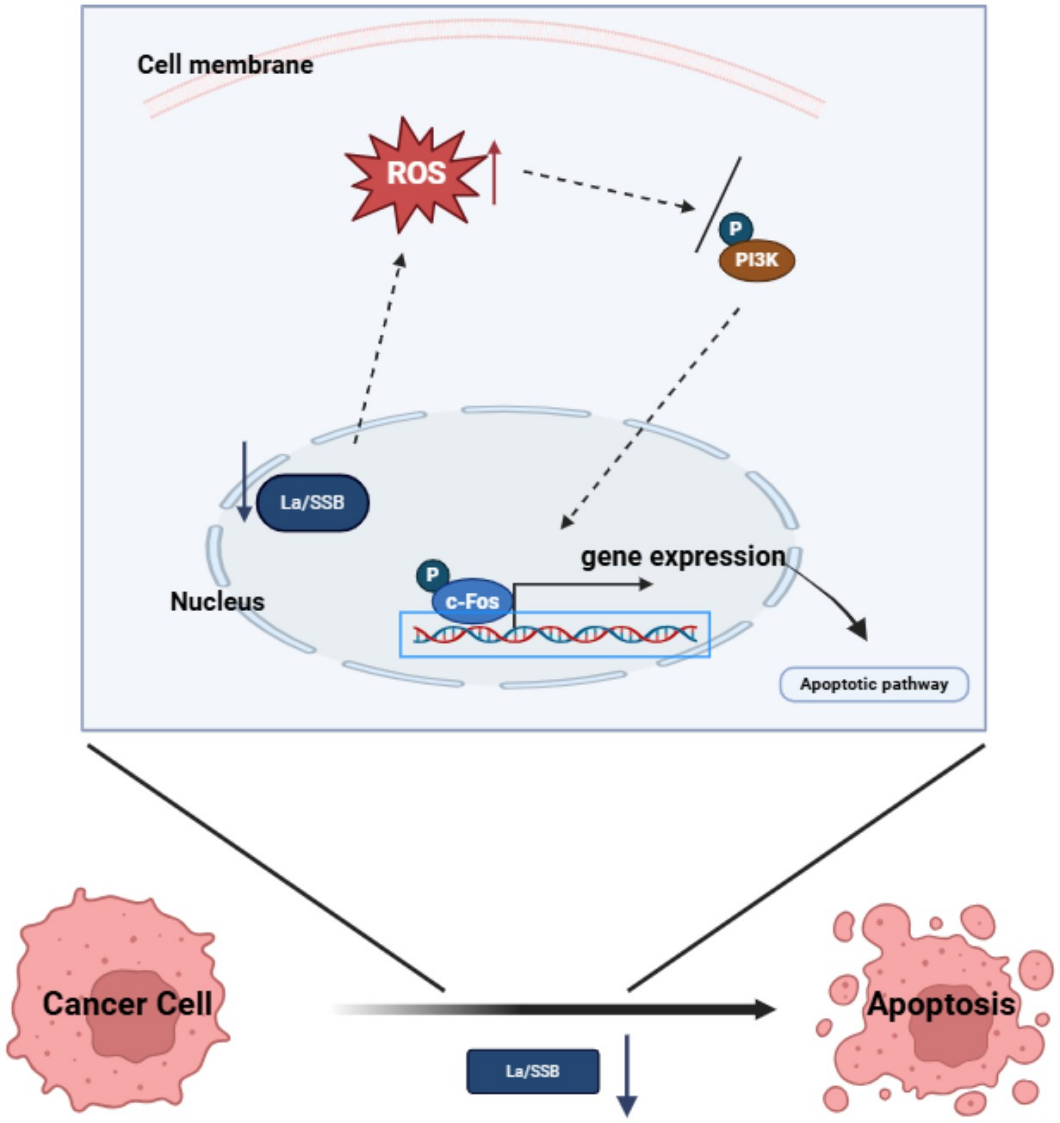
LARP3 downregulation induces HCC apoptosis through the ROS/ PI3K /c-Fos axis. After knocking down LARP3, the ROS level in HCC cells increases, which inhibits the activation of the downstream PI3K pathway, and then downregulates the expression level of c-Fos, ultimately resulting in an increase in the level of cell apoptosis and inhibiting the occurrence and development of HCC.

The application of gene sequencing technology has revealed the mutant genes and molecular markers related to the occurrence of HCC, providing a basis for early diagnosis and precise treatment [31]. Immunotherapy, especially immune checkpoint inhibitors, has shown potential in the treatment of HCC [32]. In addition, certain achievements have been made in the research and development of targeted drugs for specific targets [31]. However, in the treatment of HCC, there are many limitations. Firstly, the heterogeneity of the tumor makes its precise treatment face challenges. The cancer cells of different patients may have different biological characteristics, resulting in insensitivity or drug resistance to some treatment methods [33]. Secondly, although existing treatment means such as surgery, chemotherapy, and radiotherapy can control the tumor to a certain extent, it is still difficult to completely eradicate the cells, and the risk of recurrence is high [34]. In addition, some treatment methods may cause serious side effects, such as damage to normal tissues, affecting the quality of life and treatment tolerance of patients. For advanced HCC patients, the current treatment methods often have limited effects and it is difficult to achieve long-term survival [35]. These treatment limitations highlight the necessity of further in-depth research and exploration of new treatment strategies to improve the treatment effect of HCC and the survival prognosis of patients. In this study, the research group found through bioinformatics analysis that LARP3 is highly expressed in HCC patients and is associated with a poor prognosis. Through phenotypic studies of cell proliferation, migration and invasion, the research group found that knockdown of LARP3 will lead to inhibition of HCC proliferation, migration and invasion, and at the same time, the ROS and apoptosis levels of HCC cells increase significantly. Analyses by biological techniques such as RNA-seq, flow cytometry, RT-qPCR and Western Blot show that knockdown of LARP3 ultimately leads to apoptosis of HCC cells by upregulating the level of ROS, inhibiting the phosphorylation of PI3K and downregulating the expression of c-Fos. This research result indicates that the LARP3 gene may play an important role in the process of cell apoptosis (Fig 8).

ROS is an important mediator of cellular oxidative stress and plays various roles in the occurrence and development of HCC. High levels of ROS may cause oxidative damage to cells and trigger stress responses. ROS may also affect the function of immune cells in the tumor environment and affect the anti-tumor immune response [36]. At the same time, ROS can act as an important signal regulatory molecule in the cell and participate in many biological processes, including metabolism, apoptosis, proliferation, etc., and its research in the field of apoptosis is quite extensive [37]. Upregulating the level of ROS may be an important mechanism by which knockdown of LARP3 leads to cell apoptosis. This study shows that the knockdown of LARP3 leads to the accumulation of ROS, which in turn accelerates the process of cell apoptosis [38]. This result is in line with previous research results and further confirms the key regulatory role played by ROS in cell apoptosis. The PI3K pathway is an important intracellular signal transduction pathway that participates in the regulation of processes such as cell proliferation and differentiation [39]. The excessive activation of the PI3K pathway is closely related to the occurrence and development of cancer [40]. Studies have shown that there is a mutual influence between ROS and the PI3K pathway. In cancer, the abnormal activation of the PI3K pathway can inhibit the occurrence of apoptosis, and then promote the survival and proliferation of tumor cells, and changes in the level of ROS may also affect the survival and metastasis of cancer cells. However, the exact relationship between ROS and the PI3K pathway and their specific roles in different diseases still need further exploration [41]. This study found that the downregulation of LARP3 inhibited the activity of PI3K/c-Fos. This result reveals the potential association between LARP3 and the PI3K pathway, revealing that inhibiting the activation of PI3K/c-Fos may be a key step in promoting apoptosis, providing a new perspective for in-depth understanding of the regulatory mechanism of apoptosis. Knockdown of LARP3 may affect cell fate by regulating the ROS/PI3K/c-Fos axis, which is of great significance for the study of the pathogenesis of HCC. The research group will further explore how LARP3 regulates the level of ROS and its direct interaction with ROS and the PI3K pathway in the next step. Future research can also focus on in-depth exploration of other members of the LARP3 family and their roles in other diseases.

## Supporting information

S1 Fig Construction of LARP3 overexpression and knockdown stable transgenic strains. A: RT-qPCR was used to detect the mRNA expression of LARP3 in normal liver cells L02 and HCC cell lines Hep3B, Li-7, SUN449, and HepG-2. B: Western blot was used to detect the protein expression of LARP3 in normal liver cells L02 and HCC cell lines Hep3B, Li-7, SUN449, and HepG-2. C: RT-qPCR was used to verify the knockdown efficiency of LARP3 in HepG-2 cells. D: Western blot was used to verify the knockdown efficiency of LARP3 in HepG-2 cells. E: RT-qPCR was used to verify the knockdown efficiency of LARP3 in Li-7 cells. F: Western blot was used to verify the knockdown efficiency of LARP3 in Li-7 cells. G: RT-qPCR was used to verify the overexpression efficiency of LARP3 in Li-7 cells. H: Western blot was used to verify the overexpression efficiency of LARP3 in Li-7 cells. The data are presented as the mean ± standard deviation and come from three independent experiments with similar results. **p <0.05, ***p <0.01, ****p <0.001, *****p <0.0001.
(TIF)

S1 Table Primer information. All primers used in this study are listed in this table, including qPCR primers for GAPDH LARP3 and c-Fos; PCR primers for overexpression of LARP3 and c-Fos; and knockdown sequences for LARP3.
(PDF)

S1 Raw images (DOCX)
