# Supplementary figures and images for: LARP3 inhibits the apoptosis of hepatocellular carcinoma via the ROS/PI3K/c-Fos axis
Source: PLoS One. 2025 Jan 17;20(1):e0317454. doi: 10.1371/journal.pone.0317454 (PMC11741638; doi:10.1371/journal.pone.0317454)

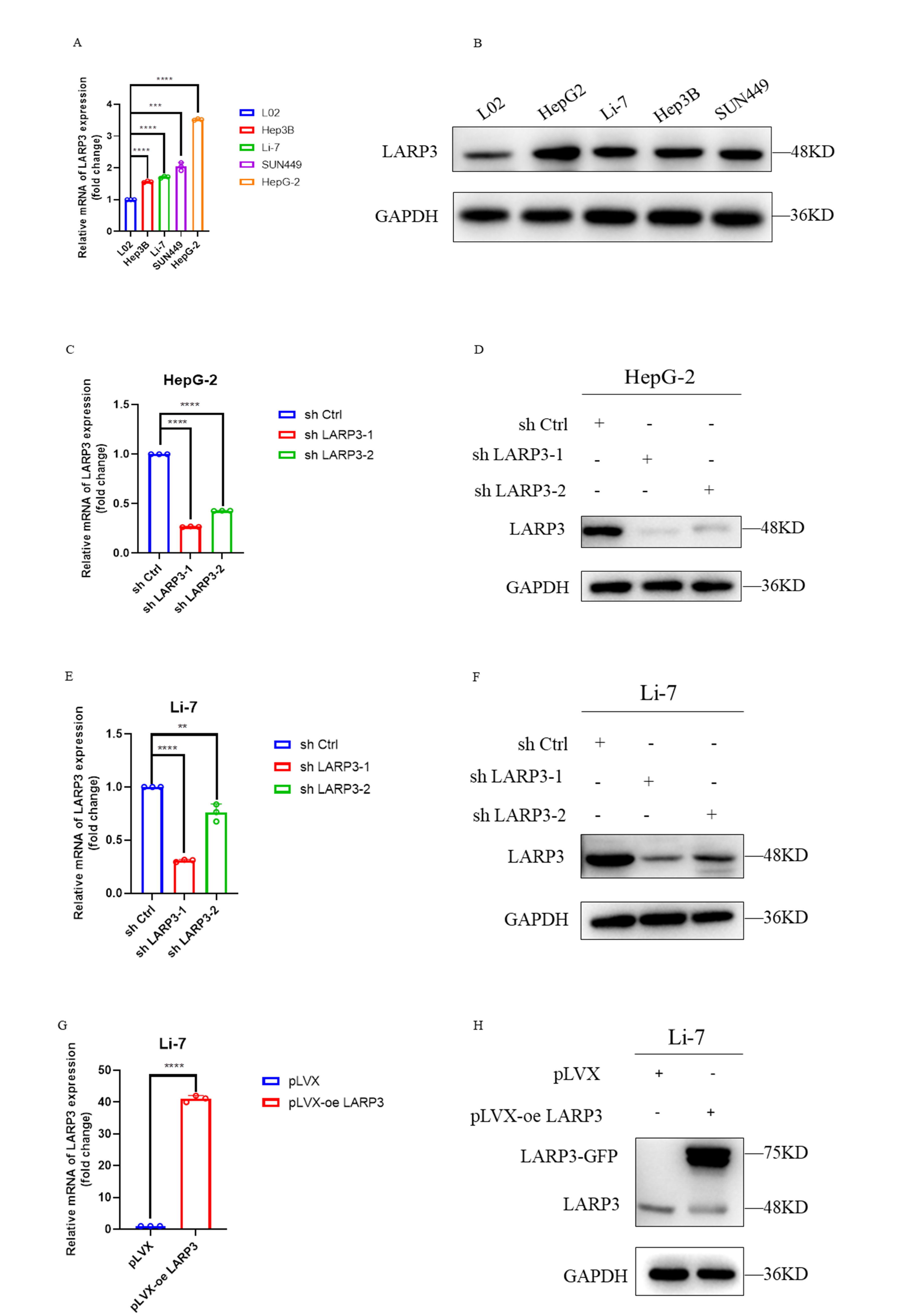

Supplement: S1 Fig — A: RT-qPCR was used to detect the mRNA expression of LARP3 in normal liver cells L02 and HCC cell lines Hep3B, Li-7, SUN449, and HepG-2. B: Western blot was used to detect the protein expression of LARP3 in normal liver cells L02 and HCC cell lines Hep3B, Li-7, SUN449, and HepG-2. C: RT-qPCR was used to verify the knockdown efficiency of LARP3 in HepG-2 cells. D: Western blot was used to verify the knockdown efficiency of LARP3 in HepG-2 cells. E: RT-qPCR was used to verify the knockdown efficiency of LARP3 in Li-7 cells. F: Western blot was used to verify the knockdown efficiency of LARP3 in Li-7 cells. G: RT-qPCR was used to verify the overexpression efficiency of LARP3 in Li-7 cells. H: Western blot was used to verify the overexpression efficiency of LARP3 in Li-7 cells. The data are presented as the mean ± standard deviation and come from three independent experiments with similar results. **p <0.05, ***p <0.01, ****p <0.001, *****p <0.0001. (TIF) [file pone.0317454.s001.tif]

**Supplementary Figure 1：**


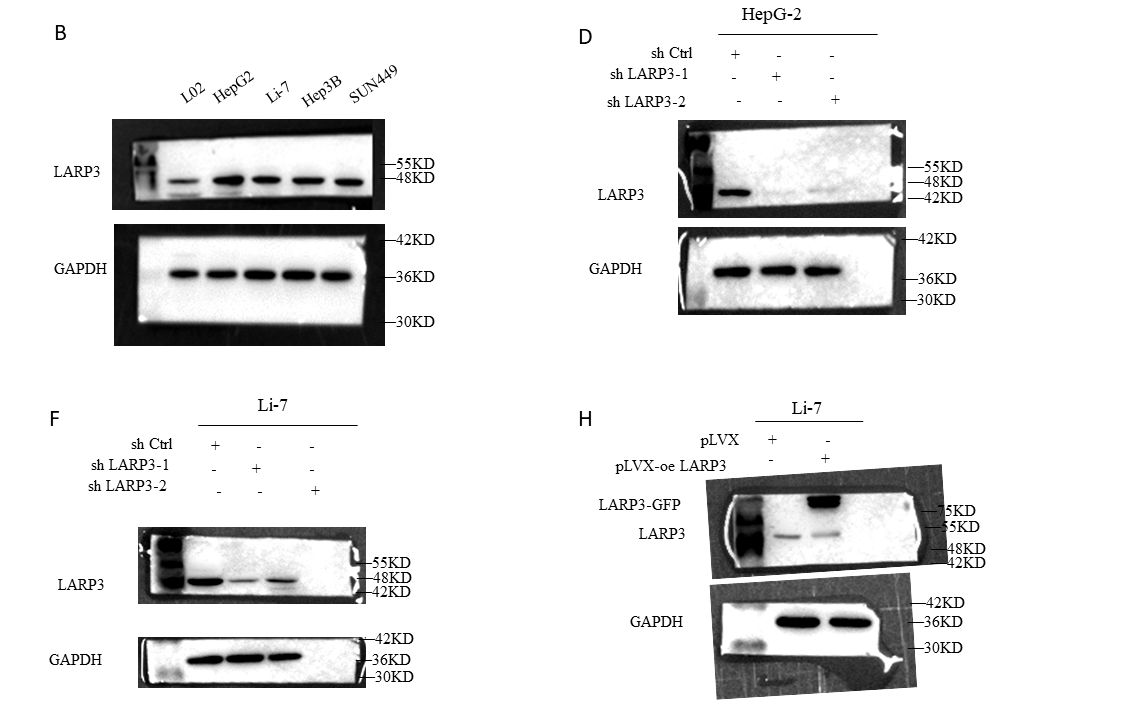


**Figure 3：**

**
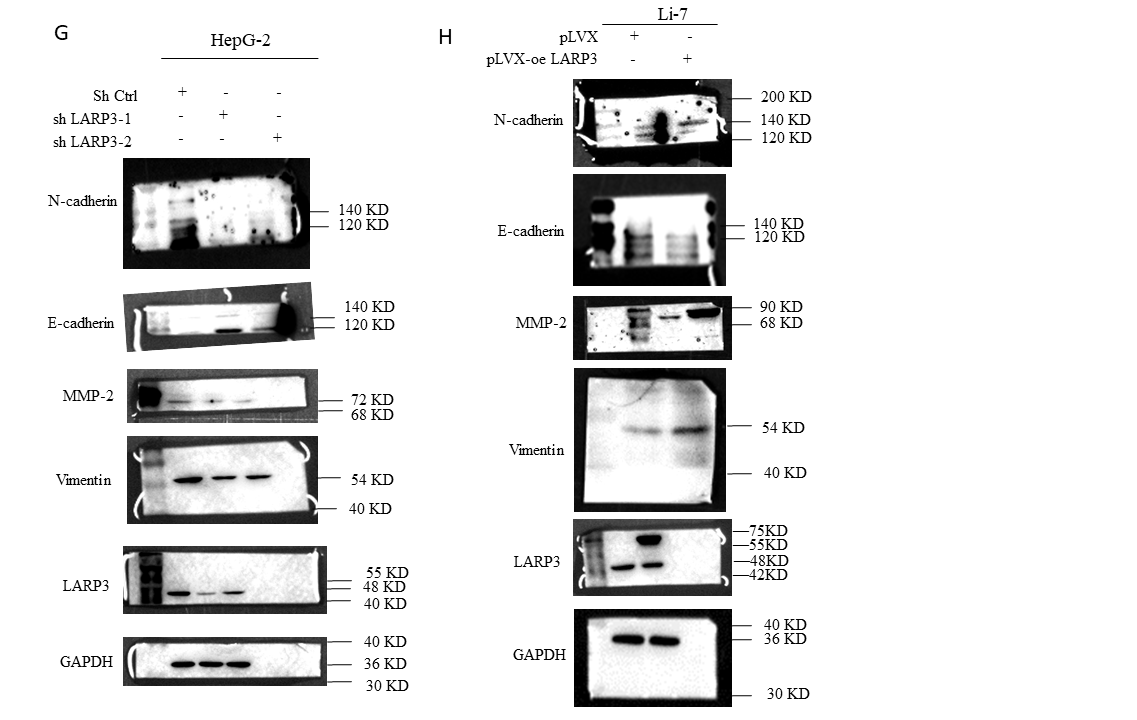
**

**Figure 4：**


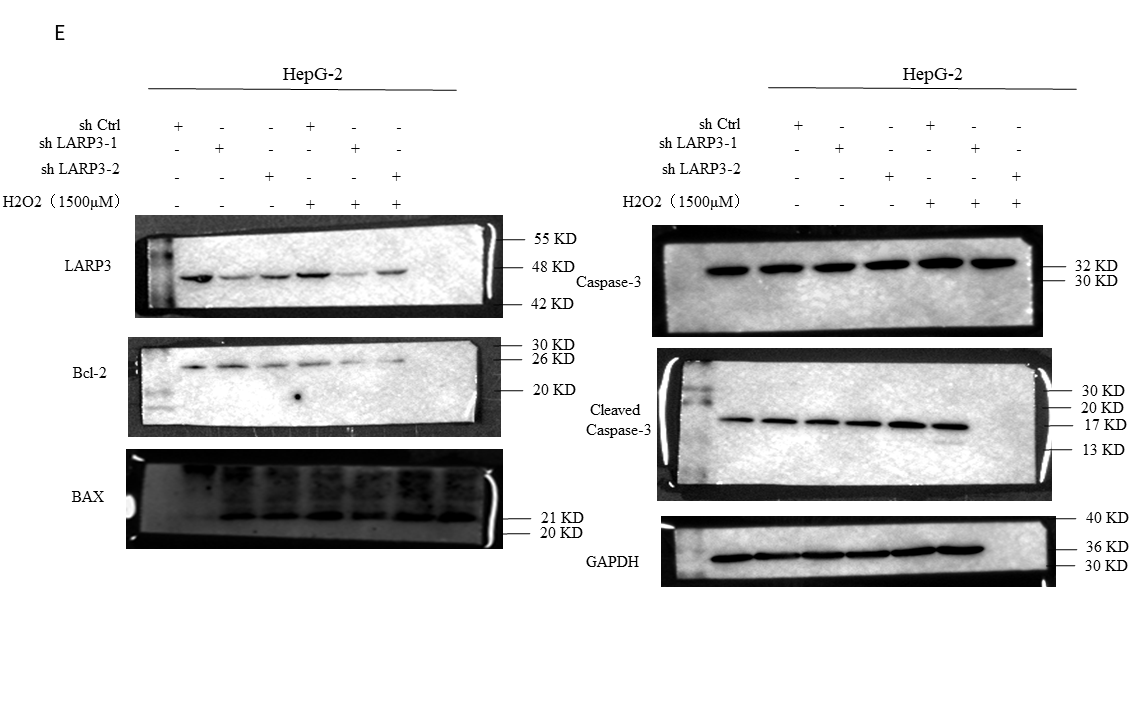


**Figure 5：**

**
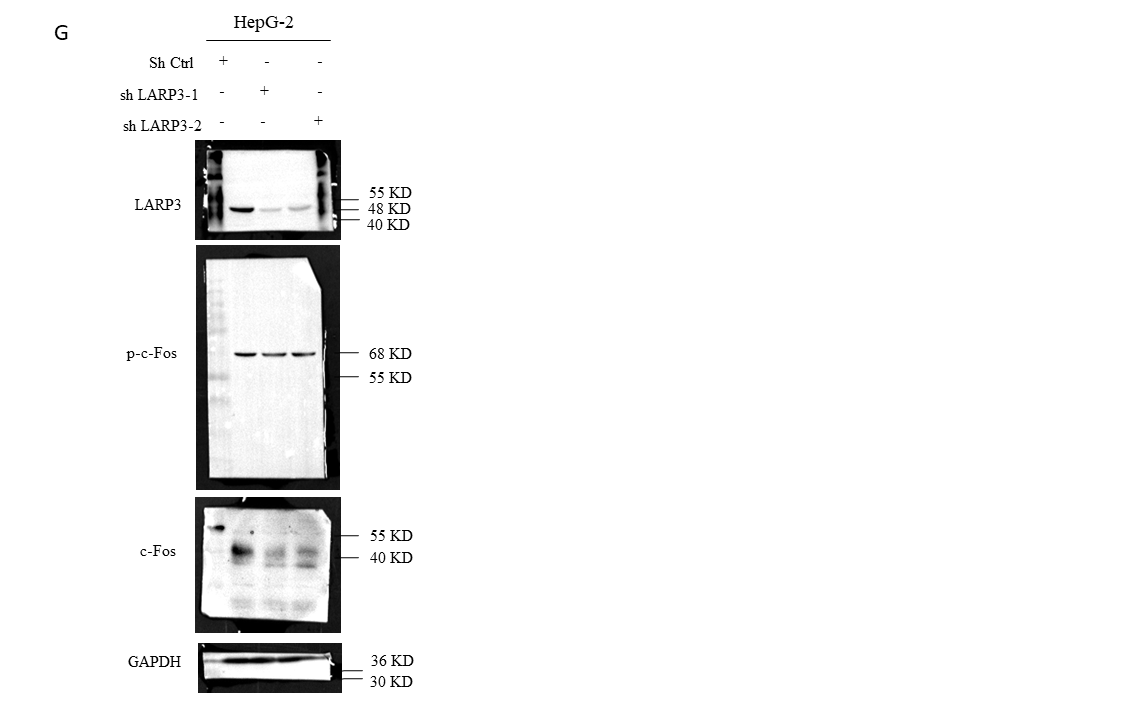
**

**Figure 6：**

**
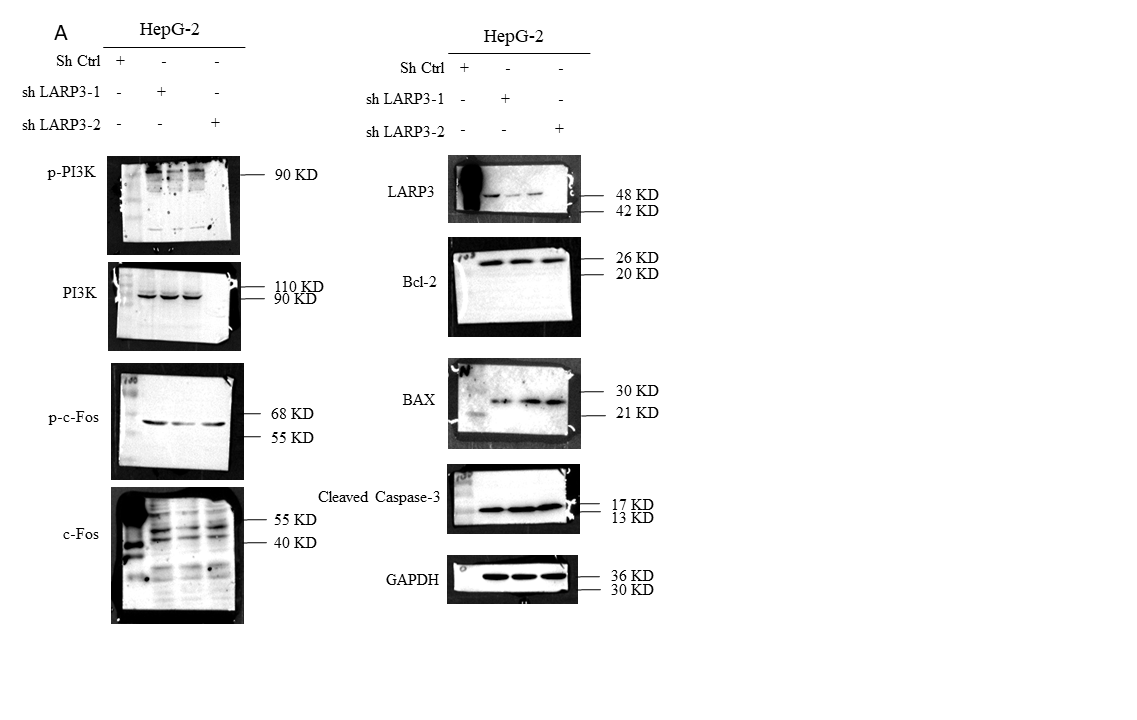
**

**
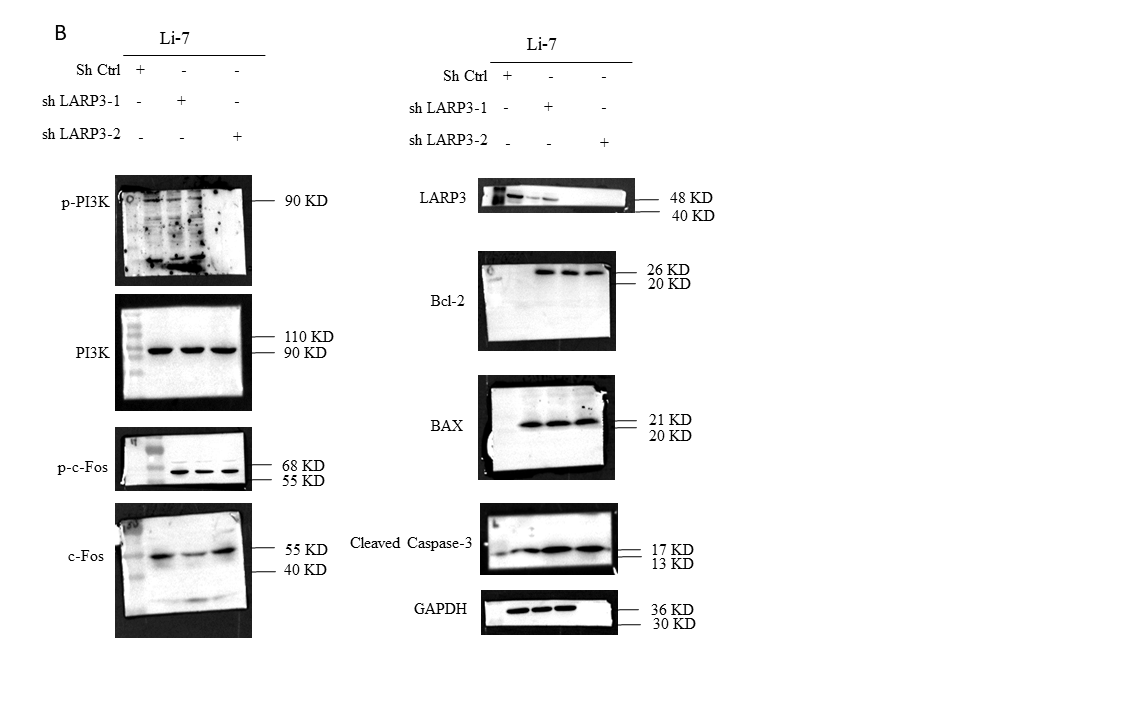
**

**
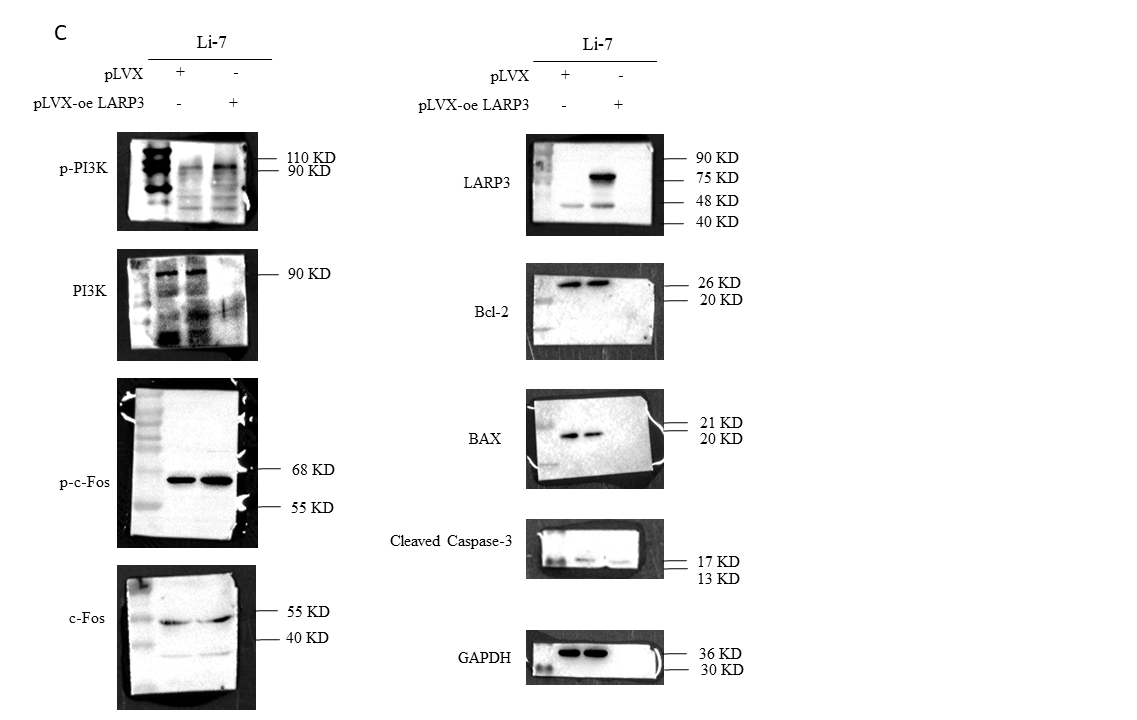
**


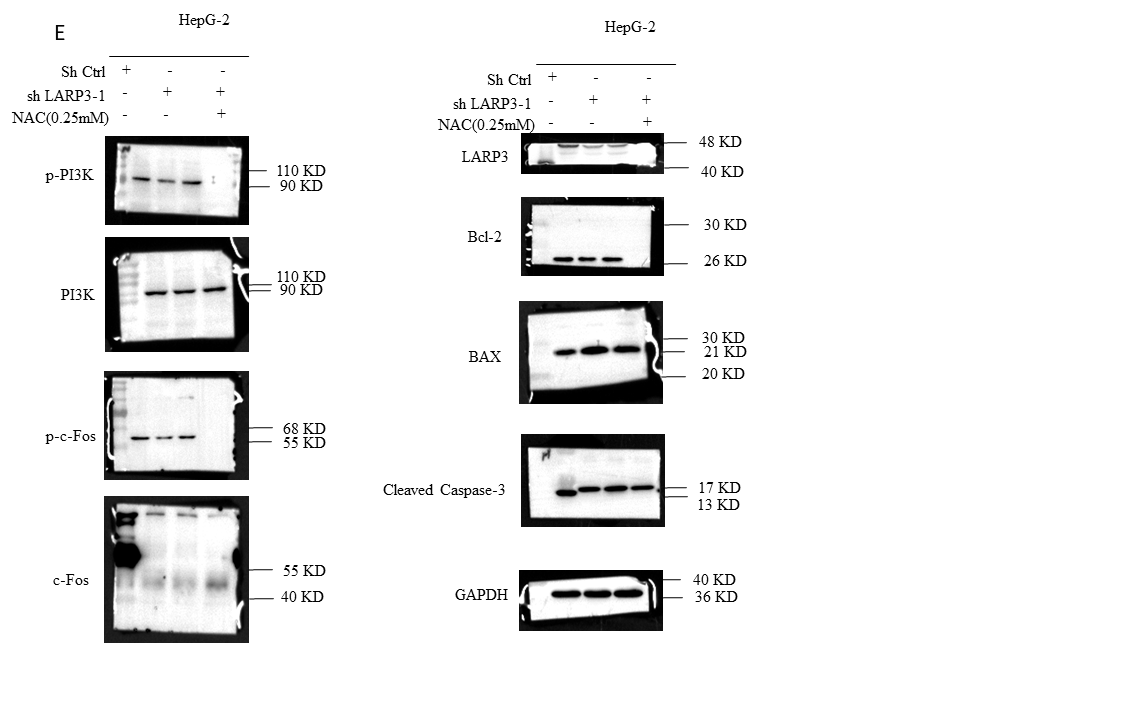


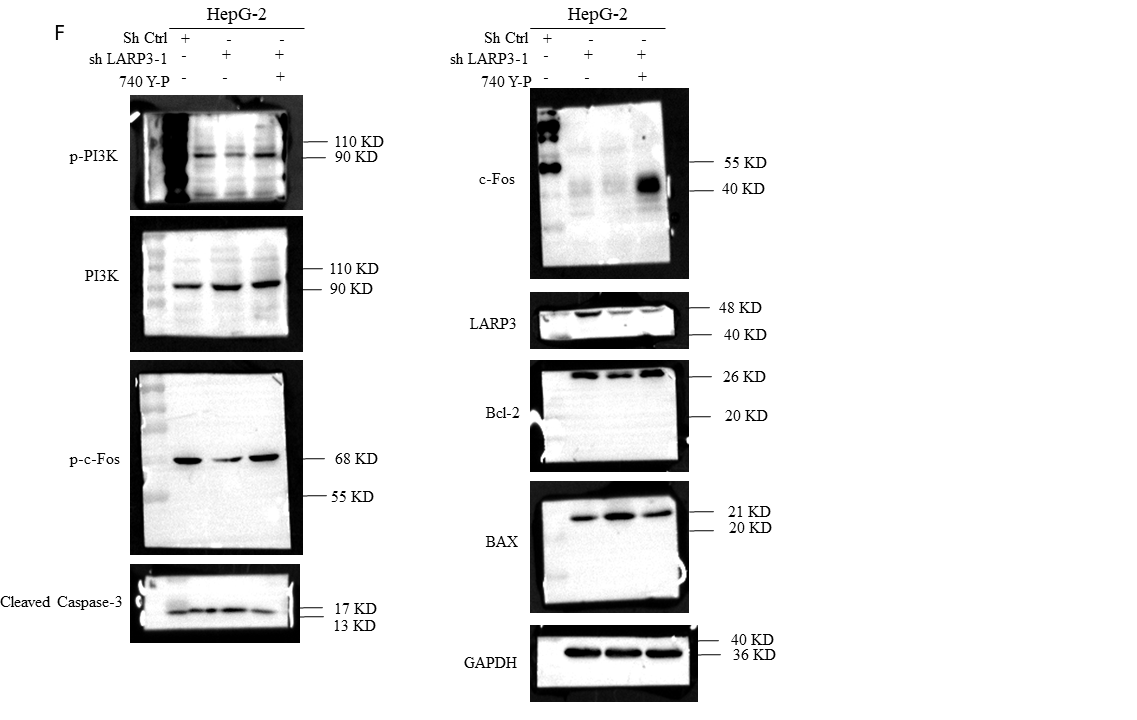


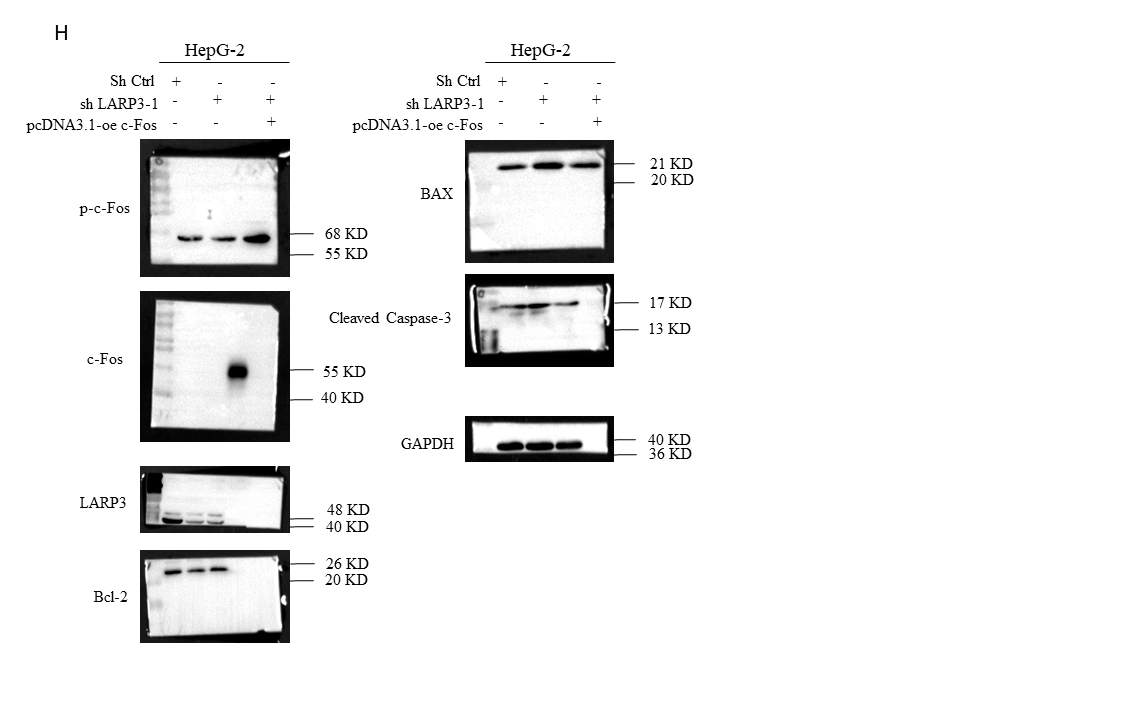

Supplement: S1 Raw images — (DOCX) [file pone.0317454.s003.docx]
