# Supplementary material for: LARP3 inhibits the apoptosis of hepatocellular carcinoma via the ROS/PI3K/c-Fos axis
Source: PLoS One. 2025 Jan 17;20(1):e0317454. doi: 10.1371/journal.pone.0317454 (PMC11741638; doi:10.1371/journal.pone.0317454)
Supplement: S1 Table — All primers used in this study are listed in this table, including qPCR primers for GAPDH LARP3 and c-Fos; PCR primers for overexpression of LARP3 and c-Fos; and knockdown sequences for LARP3. (PDF) [file pone.0317454.s002.pdf]

**Supplementary Table1:**

**Primer information:** All primers used in this study are listed in this table, including qPCR primers for GAPDH、LARP3 and c-Fos; PCR primers for overexpression of LARP3 and c-Fos; and knockdown sequences for LARP3.

| Gene name | Forward Primer | Reverse Primer |
|---|---|---|
| Q-GAPDH | GGAGCGAGATCCCTCCAAAAT | GGCTGTTGTCATACTTCTCAGG |
| LARP3 | GCCTCGAGATGGCTGAAAATG<br>TGATAATGAAAAGA | CCGGATCCCTACTGGTCTCCAGCACC ATT |
| c-Fos | TGGTACCGAGCTCGGATCCAT<br>GATGTTCTCGGGCTTCAACGC | AGGGCCCTCTAGACTCGAGTCACAGG GCCAGCAGCGTG |
| Q-c-Fos | GGCAAGGTGGAACAGTTATC | CTGCAAAGCAGACTTCTCAT |
| Q-LARP3 | GGAAAGGGTAATAAAGCTGCCC | CAGGTCCAGTTGCACCATTTT |
| sh LARP3-1 | CCGGGCTGAAATGAAATCTCTAGAACTCG AGTTCTAGAGATTTCATTTCAGCTTTTTG | AATTCAAAAAGCTGAAATGAAATCTCTAG AACTCGAGTTCTAGAGATTTCATTTCAGC |
| sh LARP3-2 | CCGGCCAAGGCAGAACTCATGGAAACTC GAGTTTCCATGAGTTCTGCCTTGGTTTTTG | AATTCAAAAACCAAGGCAGAACTCATGGA AACTCGAGTTTCCATGAGTTCTGCCTTGG |
